# Relationship between birth weight and ambient temperature during pregnancy in a cross-sectional study of the residents of Suzhou, China

**DOI:** 10.3389/fpubh.2023.1056849

**Published:** 2023-05-04

**Authors:** Yi Ding, Hui Zhou, Min Tong, Xiaofang Chen, Qian Zhao, Yuqin Ma, Lei Wu

**Affiliations:** ^1^Department of Preventive Medicine, College of Clinical Medicine, Suzhou Vocational Health College, Suzhou, Jiangsu, China; ^2^Disease Control Center of Suzhou Industrial Park, Suzhou, Jiangsu, China

**Keywords:** birth weight, pregnancy, temperature, trimester, cross study

## Abstract

**Objective:**

The association between birth weight and ambient temperature during pregnancy remains inconclusive, and data from Chinese populations are scarce. We conducted a cross-sectional study to investigate the association between birth weight and ambient temperature during pregnancy among the residents of Suzhou Industrial Park, Suzhou, China.

**Methods:**

Information regarding 10,903 infants born between January 2018 and December 2018 who were born at the hospitals in Suzhou Industrial Park, Jiangsu province was obtained via public birth records.

**Results:**

This study found that the ambient temperature during the first trimester of pregnancy was negatively correlated with birth weight, suggesting that elevated temperature may be related to lower birth weight. However, the ambient temperatures during the second and third trimesters of pregnancy were positively correlated with birth weight. Moreover, when the ambient temperature was below 15°C during the second trimester of pregnancy, the birth weight increased with temperature. However, when the temperature was higher than 15°C, the birth weight decreased with temperature. The relationship between ambient temperature in the third trimester and birth weight presented an inverted “U” curve. When the ambient temperature was lower than 20°C, the birth weight increased with ambient temperature, but when the ambient temperature was higher than 20°C, the increase of ambient temperature showed no significant relationship with the increase of birth weight.

**Conclusion:**

The ambient temperature was correlated with birth weight. The ambient temperature during the first trimester of pregnancy was negatively correlated with birth weight. The relationship between ambient temperature in the third trimester and birth weight presented an inverted “U” curve.

## Introduction

Birth weight is an important indicator for evaluating fetal growth and development, which is related to early cognition, metabolism, and risk of cardiovascular disease during life (1). It is also one of the important factors influencing the mortality rate of newborns (2). Moreover, Birth weight is associated with the risk of many chronic disease, such as hypertension, cardiovascular disease, and diabetes in adulthood. Epidemiological studies show that children born with body weight that is small for gestational age (SGA) are at a greater risk of primary hypertension (3, 4). Liang et al. (5) reported that there are non-linear inverse associations between birth weight and CVD risk, with a threshold of 3.41–3.79 kg for the lowest risk, and low birth weight may interact with adult obesity to increase the risk of CHD and heart failure. A recently published birth cohort study indicated a correlation between birth weight and brain volume in the elderly above 70 years of age (6). Lilja et al. (7) found that an inverse association between birth weight and the risk of adult stroke, IS, and ICH independent of young adult BMI, suggest that low birth weight should be included in assessments of stroke risk in adults. The above research suggests that birth weight, as a common body measurement index, has great significance, but it is often ignored.

The known influencing factors of birth weight include race, socioeconomic situation, maternal energy intake during pregnancy, and maternal weight gain during pregnancy, maternal body size, and maternal disease status, maternal nutrition before pregnancy, gestational weeks, and maternal smoking history. Numerous studies have focused on the effects of seasonal or ambient temperature on birth weight, although the results have been inconsistent. Some investigators have found seasonal variations in birth weight (possibly attributed to seasonal variations in ambient temperature), while others have not (8–13). Therefore, this study investigated the birth weight of newborns registered in the Maternal and Child Health System in Suzhou Industrial Park in 2018 in order to examine the relationship between birth weight and ambient temperature during pregnancy.

## Materials and methods

### Subjects

Information regarding 10,903 infants born between January 2018 and December 2018 who were born at the hospitals in Suzhou Industrial Park, Jiangsu province was obtained via public birth records. Pregnant women in our study are all permanent residents. The establishment of cards during pregnancy, prenatal examination, post-natal visit, and child health care of the subjects were completed in the local hospital. The information included date of birth, sex, birth weight (g), and gestational age (weeks). Information for infants who had died since the time of birth was not included. Each participant signed an informed consent form at the interview. According to the definition of WHO, infants whose birth weight is < 2,500 g are called low birth weight infants.

### Methods

Meteorological data were collected from China Meteorological Data Network including daily average temperature (°C) and daily average relative humidity (%) in Suzhou from March 20, 2017 to March 27, 2018. The data of Suzhou Air Quality Index (Daily Average) are from the National Environmental Monitoring Station. The baby weight measuring instrument is used for birth weight measurement. The unit is Kg, accurate to 2 decimal places, and the measuring accuracy is ±10 g. The measuring instrument is sent to the measurement department for calibration every year. The average temperature, relative humidity, and air quality index of early pregnancy (the last menstruation to the 12th week of pregnancy), middle pregnancy (the 13th week to the 27th week of pregnancy) and late pregnancy (the 28th week to the delivery) were calculated according to the daily average of the air quality index in Suzhou.

### Statistics

The clinical characteristics of the continuous variables were expressed as the mean ± SD and were tested using a two-sample *t-*test or ANOVA. A value of *P* < 0.05 for two-sided tests was considered statistically significant. A generalized linear model and a logistic regression model were used respectively to evaluate the effects of ambient temperature during different trimesters on the birth weight of newborns. Model 1: Adjust maternal age, pregnancy order and baby sex. Based on Model 1, the gestational age was adjusted in Model 2. Based on Model 2, environmental relative humidity and air quality index are adjusted in Model 3. A natural cubic spline with three degrees of freedom were defined for the environmental mean temperature and relative humidity in the generalized linear model. The exposure-effect curves of the environmental mean temperature and birth weight in different pregnancies were fitted. Data analyses were conducted using the SPSS Statistics 21.

## Results

### Baseline information of the study subjects

The basic information of pregnant women and newborns in the Suzhou Industrial Park 10,903 is shown in Table 1. Among the newborns with normal birth weight, significant differences in birth weight were found between mothers aged < 25 years, aged 25–30 years and aged > 30 years (*p* = 0.013), and mothers aged > 30 years had newborns with the highest birth weight. Moreover, significant differences in birth weight were found between women with 1, 2 or more pregnancies (*p* < 0.001), and women with more pregnancies had a higher newborn weight. Significant differences in birth weight were also found between newborn genders (*p* < 0.001), and males had higher birth weight than females. Furthermore, significant differences in birth weight were found between different modes of delivery (natural labor vs. cesarean section) (*p* < 0.001), and the birth weight was higher in cesarean section. Significant differences in birth weight were also found between the newborns with gestational weeks < 37, 37–41, and > 41 weeks (*p* < 0.001), and the newborns with longer gestational weeks had higher birth weight. Among the low-birth-weight newborns, no significant difference was found in maternal ages, gestational weeks, infant gender, or delivery mode (*p* > 0.05). There was a significant difference between the newborns with gestational weeks < 37 and 37–41 weeks (*p* < 0.001), and newborns with longer gestational weeks had a higher birth weight.

**Table 1:** Basic information of pregnant women and newborns.

**Characteristics**	***N***	**Birth weight (*****n*** = **10,427)**			**Low birth weight (*****n*** = **476)**		
		**Mean** ±**SD**	**F/t**	***P***	**Number**	**F/t**	***P***
Maternal age (years)			4.32	0.013		0.967	0.381
< 25	1,692	3376.14 ± 394.85			66	2014.12 ± 454.59	
25~30	5,020	3396.21 ± 395.01			214	2108.88 ± 413.11	
>30	3,715	3410.09 ± 402.75			196	2060.26 ± 427.91	
No. of pregnancies			30.1	< 0.001		2.398	0.092
1	4,896	3361.12 ± 389.59			253	2085.30 ± 428.95	
2	5,262	3424.77 ± 401.17			201	2052.79 ± 433.71	
3~	269	3450.52 ± 438.22			22	2259.09 ± 216.93	
Infant gender			133	< 0.001		0.422	0.516
Male	5,502	3440.17 ± 400.44			223	2093.09 ± 430.75	
Female	4,925	3350.67 ± 389.66			253	2067.71 ± 420.56	
Delivery mode			14.9	< 0.001		0.48	0.489
Natural labor	6,611	3386.97 ± 377.85			185	2096.54 ± 444.19	
Cesarean section	3,816	3417.67 ± 429.75			291	2068.83 ± 412.93	
Gestational weeks			463	< 0.001		39.02	< 0.001
< 37	939	3038.35 ± 339.88			408	2031.79 ± 438.05	
37~	9,439	3432.67 ± 385.22			68	2366.47 ± 134.58	
>41	49	3589.80 ± 383.03			0	-	
Total	10,427				476		

### Ambient monitoring

The daily mean temperature, daily mean relative humidity, and air quality index of pregnant women are shown in Table 2. The dispersion of daily mean temperature in the third trimester was greater than that in the first and second trimesters of pregnancy. The air quality indices in all three trimesters were good. There were significant differences in the daily mean temperature, daily mean relative humidity, and air quality index between the three trimesters.

**Table 2:** Ambient characteristics of pregnant women.

**Ambient database**	**Mean ±SD**	**T12, p1T13, p2T23, p3**	**Minimum**	**P_25_**	**P_50_**	**P_75_**	**Maximum**
Daily mean temperature							
First trimester of pregnancy:	18.20 ± 8.15	6.073, < 0.001	5.4	10.36	18.98	25.98	29.10
Second trimester of pregnancy:	17.52 ± 7.76	2.997, 0.003	6.0	9.88	17.49	25.42	28.33
Third trimester of pregnancy:	17.19 ± 8.19	6.567, < 0.001	1.8	9.14	17.98	24.88	30.30
Daily mean relative humidity (%)							
Second trimester of pregnancy:	75.29 ± 1.73	16.93, < 0.001	72	73.72	75.29	76.77	79
Third trimester of pregnancy:	74.93 ± 2.26	11.88, < 0.001	54	73.2	73.20	76.61	89
Air index							
First trimester of pregnancy:	84.72 ± 10.55	4.761, < 0.001	66	74.55	85.87	92.31	105
Second trimester of pregnancy:	83.93 ± 9.99	0.276, 0.783	68	74.43	85.06	91.15	103
Third trimester of pregnancy:	84.77 ± 11.95	5.034, < 0.001	54	74.46	84.16	93.59	133

### Effect of ambient temperature during pregnancy on birth weight

The results of logistic regression analysis between ambient temperature during pregnancy and birth weight in 10,471 newborns with normal birth weight are shown in Table 3. After adjusting for maternal age, parity and infant gender, Model 1 showed no significant correlation between ambient temperatures in the first, second and third trimesters of pregnancy and birth weight. After adjusting the gestational weeks based on Model 1, Model 2 also showed no correlation between ambient temperature during pregnancy and birth weight. After further adjusting the ambient relative humidity and air quality index based on Model 2, Model 3 showed a significant negative correlation between the ambient temperature in the first trimester of pregnancy and birth weight (*p* < 0.001), wherein the birth weight decreased by 3.134 g (1.271–4.997 g) for every 1°C increases in temperature. There was no significant correlation between ambient temperatures in the second and third trimesters of pregnancy and birth weight.

**Table 3:** Correlation between ambient temperature (°C) during pregnancy and normal birth weight of newborns.

**Trimester of pregnancy**	**Model 1**		**Model 2**		**Model 3**	
	**b (95% CI)**	***P***	**b (95% CI)**	***P***	**b (95% CI)**	***P***
First trimester of pregnancy:	0.249 (-0.871 to 1.369)	0.663	0.345(-0.744 to 1.433)	0.535	−3.134(-4.997 to−1.271)	0.001
Second trimester of pregnancy:	0.004 (-1.153 to 1.161)	0.995	−0.084(-1.208 to 1.040)	0.883	−0.249(-2.017 to 1.519)	0.782
Third trimester of pregnancy:	0.278 (-0.801 to 1.357)	0.614	0.155(-0.898 to 1.208)	0.774	0.302(-1.168 to 1.772)	0.688

Model 1 was adjusted for maternal age, parity and infant gender. Model 2 was adjusted for gestational weeks based on Model 1. Model 3 was adjusted for the relative humidity and air quality index based on Model 2.

The results of logistic regression analysis between ambient temperature during pregnancy and birth weight in 476 newborns with low-birth weight are shown in Table 4. After adjusting for maternal age, parity, and infant gender, Model 1 showed no significant correlation between ambient temperature in the first, second and third trimesters of pregnancy and birth weight. There was no significant correlation between ambient temperature and during pregnancy and newborn birth weight whether further adjusting gestational weeks on Model 2 or further adjusting temperature ambient relative humidity or air quality index on Model 3.

**Table 4:** Correlation between ambient temperature (°C) during pregnancy and low birth weight of newborns.

**Trimester of pregnancy**	**Model 1**		**Model 2**		**Model 3**	
First trimester	1.007 (0.994–1.019)	0.2970	1.004 (0.986–1.021)	0.6981	0.994 (0.965–1.025)	0.7030
Second trimester	0.990 (0.978–1.003)	0.1285	0.992 (0.973–1.010)	0.3757	1.005 (0.975–1.035)	0.7535
Third trimester	0.989 (0.977–1.001)	0.0615	0.999 (0.982–1.016)	0.9020	0.998 (0.976–1.022)	0.8963

Model 1 was adjusted for maternal age, parity and infant gender. Model 2 was adjusted for gestational weeks based on Model 1. Model 3 was adjusted for the relative humidity and air quality index based on Model 2.

**Figure 1:**
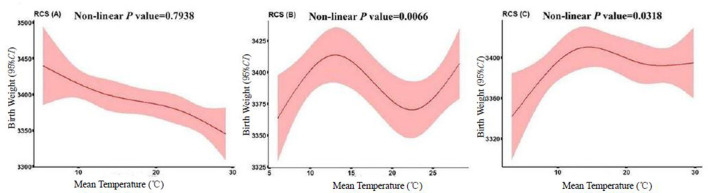
Dose-response relationship between ambient temperature during pregnancy and normal birth weight of 10,471 newborns. Correlation between ambient temperature during pregnancy and birth weight of newborns estimated using the generalized non-linear model. The model controls for maternal age, education level, number of pregnancies, and mode of delivery, gestational weeks, infant gender, relative humidity, and air quality index. From left to right: first trimester of pregnancy **(A)**; second trimester of pregnancy **(B)**; third trimester of pregnancy **(C)**. The light pink region is the 95% CI.

The relationship between birth weight and ambient temperatures during first, second and third trimesters of pregnancy was analyzed by the generalized non-linear curve in 10,471 pregnant women and newborns. The results showed no non-linear relationship between birth weight and ambient temperature in the first trimester of pregnancy. A significant non-linear correlation was found between birth weight and ambient temperatures in the second and third trimesters of pregnancy, with *p*-values of correlation coefficients of 0.0066 and 0.0318, respectively (Figure 1).

## Discussion

This study found that the ambient temperature during the first trimester of pregnancy was negatively correlated with birth weight, suggesting that elevated temperature may be related to lower birth weight. However, the ambient temperatures during the second and third trimesters of pregnancy were positively correlated with birth weight. Moreover, when the ambient temperature was below 15°C during the second trimester of pregnancy, the birth weight increased with temperature. However, when the temperature was higher than 15°C, the birth weight decreased with temperature. The relationship between ambient temperature in the third trimester and birth weight presented an inverted “U” curve. When the ambient temperature was lower than 20°C, the birth weight increased with ambient temperature, but when the ambient temperature was higher than 20°C, the increase of ambient temperature showed no significant relationship with the increase of birth weight. The results of this study were consistent with several large cohort studies worldwide. Lawlor et al. (14) studied the Aberdeen children cohort in the 1950's and found that the mean outdoor temperature in the first trimester of pregnancy was negatively correlated with birth weight, while the mean outdoor temperature in the third trimester was positively correlated with birth weight. Some retrospective cohort studies (15–19) and ecological studies (20–25) have found that hotter weather was associated with lower birth weight. In contrast, other studies have found that hot weather was associated with higher birth weight (26), or birth weight decreased with decrease in temperature (27, 28). A recent large cohort study reported that temperatures both above and below the mean temperature were correlated with reduced full-term birth weight (although the decrease below the mean temperature was small), suggesting an inverted U-shaped relationship (29). Similarly, another recent cohort study found that low full-term birth weight was correlated with high and low ambient temperatures (30). One study found that the correlation between body weight and ambient temperature may be associated with exposure gestational weeks (14). However, some studies reported no correlation between the temperature during pregnancy and birth weight or low birth weight (31–33).

An animal study indicated that chronic heat stress during pregnancy may reduce blood flow in the uterus and umbilical cord and placental weight, resulting in lower birth weight of many species (24). However, the correlation of the potential mechanism in humans remains unclear. Some studies have reported the correlation between temperature change and inflammatory markers. Although many of them have observed evidence of inflammation with decrease in temperature (21, 34–36), other studies have suggested that lower temperature reduces the production of inflammatory markers (37) or inflammatory markers increase with increase in temperature (38). However, these results suggested that temperature variation may correlate with inflammatory markers. Inflammatory mechanisms may play a role in the correlation between weather and fetal growth. Besides, heat stress can induce oxidative stress, and placental oxidative stress may be one of the causes of intrauterine growth restriction (39–41). However, these potential mechanisms cannot explain the possible impact of temperature changes on birth weight as a concept, independent of the mean temperature. Continuous temperature changes may interfere with the body's ability to recover from heat stress-induced inflammatory changes or blood flow patterns. Moreover, given the changes in the cardiovascular system and other physiological processes during pregnancy (42), pregnant women may have lower thermoregulatory capacity and adaptability to rapid temperature changes.

This study had some limitations. First, the indoor and outdoor activity times and real-time ambient temperatures of pregnant women were not monitored, which may have led to inaccurate estimation of real exposure temperature. Second, confounding factors, such as maternal living habits and nutritional status, were unavailable. Third, our study didn't analyze the relationship between humidity and newborn birth weight. Humidity is known to reduce the body's ability to release heat, its potential independent effects on health are unclear (24, 25). These limitations may have led to bias. However, this study had a large sample size, and the records of birth weight and maternal indicators were directly obtained from the regional information system, which led to high reliability and accuracy of the data. Thus, the results of this study have scientific value and clinical guiding significance.

In conclusion, our study has examined the association between the ambient temperature and birth weight based on normal and low birth weight population analyses. We found that the ambient temperature during the first trimester of pregnancy was negatively correlated with birth weight, suggesting that elevated temperature may be related to lower birth weight. Moreover, the ambient temperatures during the second and third trimesters of pregnancy were positively correlated with birth weight. Additionally, the relationship between ambient temperature in the third trimester and birth weight presented an inverted “U” curve. Independent replications in large sample sizes are needed to confirm the role of the ambient temperature with newborn birth weight found in this study for normal and low birth weight population.

## Data availability statement

The raw data supporting the conclusions of this article will be made available by the authors, without undue reservation.

## Author contributions

YD and LW conceived and designed the research. YD wrote the manuscript. LW, YD, MT, YM, and XC revised it critically for important intellectual content. YD and HZ performed the data analysis. All authors contributed to the interpretations of the findings, reviewed the manuscript, contributed to the article, and approved the submitted version.
